# Remote ischemic conditioning improves cognition in patients with subcortical ischemic vascular dementia

**DOI:** 10.1186/s12883-019-1435-y

**Published:** 2019-08-23

**Authors:** Zhangyuan Liao, Yali Bu, Meijie Li, Ranran Han, Nan Zhang, Junwei Hao, Wei Jiang

**Affiliations:** 1 Department of Neurology, Tianjin Medical University General Hospital, Tianjin, 300052 China; 2 Tianjin Neurological Institute, Key Laboratory of Post-neurotrauma Neuro-repair and Regeneration in Central Nervous System, Ministry of Education and Tianjin City, Tianjin, 300052 China

**Keywords:** Ischemic conditioning, Cerebral small vessel disease, Subcortical ischemic vascular dementia, Cognition, White matter lesion, Diffusion tension imaging

## Abstract

**Background:**

Subcortical ischemic vascular dementia (SIVD) is very common among the older people, but has no approved treatment. Preclinical trials show that remote ischemic conditioning (RIC) reduces recurrence of ischemic stroke. We hypothesize that RIC may also be an effective therapy for patients with SIVD.

**Methods:**

Thirty-seven consecutive SIVD cases were enrolled in this randomized control study. Eighteen RIC patients underwent five brief cycles of conditioning (bilateral upper limb compression at 200 mmHg) followed by reperfusion twice daily over 6 consecutive months. Nineteen control patients underwent the same process, but at a pressure of 60 mmHg which caused no restriction on the blood flow of the upper limb. The primary outcome measures were changes in neuropsychological assessments. The secondary outcomes included the changes in high-sensitive C-reactive protein (hs-CRP) concentration, white matter lesion volume (WMLV), diffusion tension imaging (DTI) metrics of white matter. All data were collected at baseline and follow-up.

**Results:**

A significant treatment difference favoring RIC at 6 months was observed on performance of Hopkins Verbal Learning Test-Revised (HVLT-R), Controlled Oral Word Association Test (COWAT), Trail Making Test A and B (TMT-A & TMT-B), and Judgment of Line Orientation (JLO) (*p* < 0.05). The control group did not show much improvement after the treatment, and only with a slight change in HVLT-R and TMT-R (*p* < 0.05). Covariance analysis of efficacy between the two groups suggested that RIC patients performed better on JLO than control patients at the 6-month follow-up (RIC 23.10 vs. control 18.56; *p* = 0.013). Although DTI metrics were comparable, Hs-CRP levels and WMLV in RIC patients showed a declining trend.

**Conclusions:**

Over the 6-month treatment period, we found that RIC was safe and effective for improving cognitive function in SIVD patients.

**Trial registration:**

Clinical Trial Registration (http://www.clinicaltrials.gov), Unique identifier: NCT 03022149; Retrospectively registered; Date of registration: January 16, 2017.

## Background

Vascular dementia is the second most common cause of dementia after Alzheimer’s disease, comprising around 15% of dementia cases [1]. Subcortical ischemic vascular dementia (SIVD) is a major cause of vascular dementia, which is clinically homogeneous and results from small vessel disease and hypoperfusion [2]. However, unlike Alzheimer’s disease, no authorized treatments exist for SIVD to date.

One treatment that might prove useful for improving cerebral circulation is remote ischemic conditioning (RIC). In RIC, a short ischemic attack is carried out in limb or organ, thus ischemic protection is induced in the brain remotely [3]. The mechanisms in RIC are complex and interlinked, which could either promote anti-inflammatory cascades or inhibition of pro-inflammatory cytokine synthesis, and increase the resistance of cells or tissues against a subsequent, more rigorous ischemic event [4]. Transient limb ischemic conditioning, which could help to reduce proinflammatory cytokine synthesis and increase cerebral blood flow, has been suggested to be a protective treatment against recurrent stroke in cranial atherosclerotic stenosis [5, 6].

In small vessel disease, hypoperfusion as a consequence of microangiopathy can accelerate neurodegeneration, blood-brain barrier disruption, and neuroinflammation [7]. Lower cognitive performance is associated with lower microvascular perfusion [8]. Plasma high sensitive C-reactive protein (hs-CRP) and interleukin-6 (IL-6) are the most commonly studied inflammatory biomarkers in SIVD. Hs-CRP, which is an acute-phase reactant synthesized in the liver in response to IL-6, has been widely used as a vascular inflammatory biomarker [9]. Besides, white matter lesion load was inversely associated with cognitive performance [10]. Therefore, we tested whether RIC can improve the cognition in SIVD patients and its effect on pro-inflammatory mediators and white matter lesion.

## Methods

### Study design

Patients diagnosed as SIVD were enrolled in this clinical trial. Inclusion criteria [11] were as follows: (1) age 50–80 years old; (2) complaint of cognitive impairment lasting for at least 3 months; and (3) vascular dementia diagnosis, according to the criteria of the Diagnostic and Statistical Manual of Mental Disorders, Fourth Edition, with a Mini-Mental State Examination (MMSE) score 10–26; Montreal Cognitive Assessment (MOCA) score < 26; and Clinical Dementia Rating (CDR) score 1–2. All patients meeting the clinical criteria underwent brain MR imaging (MRI). The MRI inclusion criteria were as follows [12], 1) moderate to severe white matter lesions (score ≥ 2, according to the Fazekas rating scale [13]); or multiple (≥3) small supratentorial subcortical infarcts (3–20 mm in diameter); or small infarcts strategically located in the caudate nucleus, globus pallidus, or thalamus; (2) absence of hemorrhages, cortical and watershed infarcts, hydrocephalus, and white matter lesions from specific causes (e.g, multiple sclerosis). Exclusion criteria included dementia diagnosed as other causes, such as Alzheimer’s disease and Lewy body dementia; the patients cannot complete neuropsychological testing (e.g., severe aphasia, physical disabilities), or experienced new strokes within 3 months before enrollment; small vessel disease due to inheritance or inflammation; schizophrenia or a score > 17 on the Hamilton Depression Scale (HAMD); cancer; clinically significant systemic diseases (e.g., cardiovascular, respiratory); peripheral vascular disease; use of donepezil and memantine that may affect cognitive functioning; Refusal to sign informed consent.

### RIC and control treatment procedures

Participants were randomly divided into two groups according to a random number. The experimental group underwent five brief cycles of RIC (bilateral upper limb compression at 200 mmHg) for 5 min followed by reperfusion for another 5 min, which performed twice daily over 6 consecutive months. The control group underwent the same process, but at a pressure of 60 mmHg which caused no restriction on the blood flow of the upper limb. RIC was carried out by an electric inflation auto-control device (patent number ZL200820123637.X, RenQiao IPC-906D, China), which is similar to the blood pressure measurement [6]. Participants could abort the RIC treatment at any time if they did not feel well.

Special personnel unassociated with the study were responsible for randomization and allocation of RIC instruments. The patients and related research personnel, including the neuropsychiatric tests, image processing and biomarker detection, were blind to the treatment allocation.

During the entire 6-month study, both groups of patients continued taking their standard medications, including anti-platelet, anti-hypertensive, anti-diabetic, anti-homocysteine, and lipid control agents. A few patients took *Ginkgo biloba* leaves and citicoline tablets before enrollment. Because the nootropic effect is not clear, these drugs have not been discontinued mandatorily.

### Treatment compliance guarantee

The data of each treatment were recorded by the RIC device and sent via the internet to our researchers’home computer in real time. The investigators scanned the participants’ therapy compliance routinely. Only when the time and frequency per day achieved the standard, the treatment could be qualified. If abnormal conditions occured in the course of the treatment, the researcher would contact the patient or family members in time. If necessary, the patients could complete the treatment with the assistance of family members. Participants who did not complete the treatment for seven consecutive days were excluded.

### Neuropsychological testing

The primary outcome measures for assessing RIC efficacy in improving cognition were neuropsychological assessments. These assessments included five domains: memory, language, attention, executive function, and orientation. Immediate memory, delayed memory, and recognition memory were tested with the Hopkins Verbal Learning Test-Revised (HVLT-R) [14]. Language usage and category fluency were tested with the Controlled Oral Word Association Test (COWAT) [15]. Attention and executive function were tested with the Trail Making Test A and B (TMT-A and TMT-B) [16], and the Symbol Digit Modalities Test (SDMT) [17]. Visuospatial processing was examined with the Judgment of Line Orientation (JLO) [18]. Cognitive tests were administered at baseline and 6 months later.

### Measurements of inflammation

Blood was collected (5 ml) from the antecubital vein of each participant and deposited in commercially available anticoagulant-treated (ethylenediaminetetraacetic acid [EDTA]) plasma tubes. Plasma was separated from blood cells within two hours of collection and stored at − 80 °C until analysis.

Interleukin-6 (IL-6), tumor necrosis factor-alpha (TNF-α) were assessed using commercially available ELISA kits (Bio-Source Europe, Nivelles, Belgium), and Hs-CRP was measured using commercially available turbidimetric immunoassay kits (MedicalSystem Biotechnology Co., Ltd., Ningbo, China), following the manufacturer’s instructions. Other biochemical markers were measured by an automatic biochemical analyzer.

### MRI acquisition

MRI examinations of the head were done using a 3.0 T whole body system (Discovery MR750; General Electric, Milwaukee, WI, USA). Cube FLAIR (156 slices; repetition time (TR), 6000 ms; echo time (TE), 144 ms; echo train length, 200; slice thickness, 1.2 mm; in-plane resolution, 1 mm^2^) was used for lesion detection. High-resolution sagittal three-dimensional (3D) T1-weighted images were acquired using a brain volume (BRAVO) sequence (156 slices; TR, 8.14 ms; TE, 3.17 ms; inversion time (TI), 450 ms; flip angle, 12°; slice thickness, 1.2 mm; in-plane resolution, 1 mm^2^). The high-resolution images were used to calculate brain volume. Also, two-dimensional (2D) echo-planar diffusion tensor images (DTI) were acquired (48 slices; TR, 5000 ms; TE, 60.6 ms; slice thickness, 3 mm; in-plane resolution, 2 mm^2^; 50 non-collinear diffusion gradients [b = 1000 s/mm^2^]). Three non-diffusion-weighted images (b = 0 s/mm^2^) were used for measuring white matter tract integrity.

### Analysis of White matter lesions

Demarcations of interest regions and measurement of white matter lesion volume (WMLV) on cube FLAIR images were performed manually by using MRIcro software (http://www.mccauslandcenter.sc.edu/mricro/mricro/mricro.html).

### DTI analysis

We extracted the mean diffusion indices of the Whole-Brain White Matter (WBWM), White Matter Lesion (WML) and Normal-Appearing White Matter (NAWM) (WBWM minus lesion regions). DTI data were first processed along the following pipeline using the FMRIB Software Library (FSL) 5.0: (1) Eddy current correction: this step applied an affine transformation on the raw diffusion data to correct for image distortion caused by eddy current, and it corrected for misalignment between volumes caused by head motion. (2) Brain extract: voxels outside brain tissue were filtered out using the brain extract toolbox (BET) in FSL. Then, a linear least-squares fitting algorithm was carried out to fit the tensor, and the three eigenvalues, Mean Diffusivity (MD), and Fractional Anisotropy (FA) were calculated from the tensor.

Brain lesions were manually segmented from the 3D T2 FLAIR images and saved as a binary mask for each subject. To separate the three different types of diffusion indices (WBWM, WML, and NAWM), we first segmented the 3D T1 weighted images (T1-WI) into gray matter tissue, white matter tissue, and cerebrospinal fluid using a unified segmentation method carried out in SPM8 (http://www.fil.ion.ucl.ac.uk/spm/software/spm8/). Then the white matter tissue of each subject was binarized to create a whole white matter mask using a threshold of 50%. The T1WI was then affinely co-registered with the b0 images (diffusion images without exerting a gradient field). T2 FLAIR images were rigidly co-registered with the T1 images, and then were transformed into the diffusion space along with the lesion mask using the deformation parameters between T1 and b0 images. The normal white matter mask for each subject was calculated by subtracting the whole white matter mask from the white matter lesion mask. Finally, the mean diffusion metrics (FA and MD) of each mask of each subject were calculated by averaging the values of all voxels with this mask.

### Statistical analysis

Baseline data homogeneity between the two groups was analyzed with two independent samples t-test, X^2^ test, Fisher’s exact test and the Mann-Whitney U test. Data from the pre-treatment and post-treatment periods were analyzed by paired t-test to evaluate the effect of the treatments in the group. Due to the small sample size, covariance analysis was used to compare the effect of the two groups.

All analyses were done with SPSS (IBM SPSS Statistics for Windows, Version 22.0). All hypothesis tests were two-tailed, and *P* values < 0.05 were considered significant.

## Results

### Characteristics of patient population

**Fig. 1:**
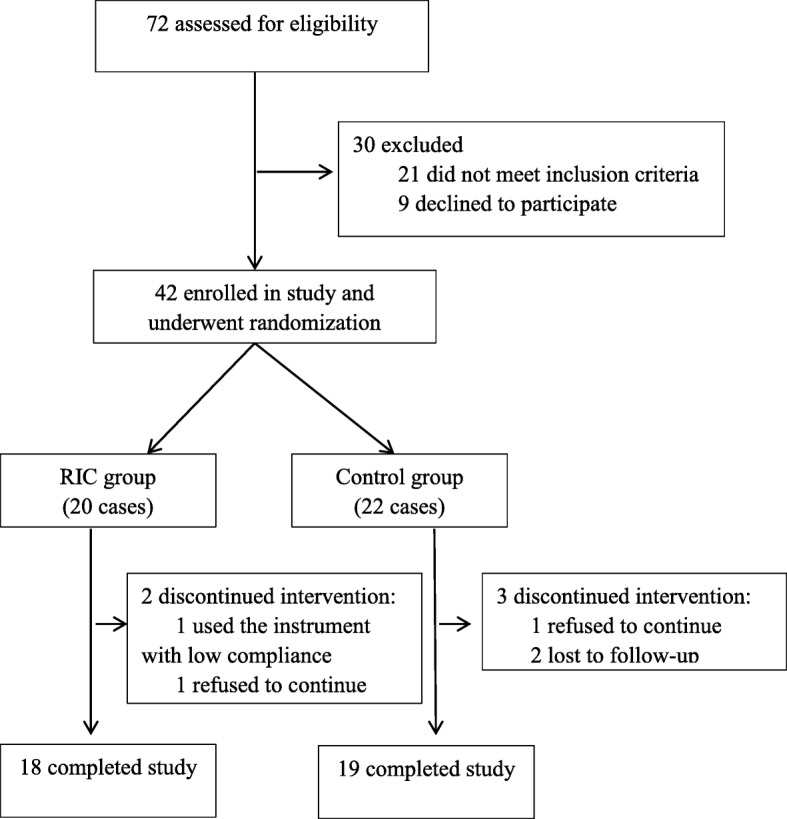
Study cohort allocation

Between January 2016 and September 2016, 72 patients were screened from the neurology outpatient department of Tianjin Medical University General Hospital. Of these, 30 were excluded because 21 failed to meet the inclusion criteria and 9 declined to participate. This left a total of 42 participants who met all inclusion criteria and were assigned to either the RIC group (*n* = 20) or the control group (*n* = 22). Five cases were excluded due to poor compliance or were lost to follow-up. Finally, 37 cases in the two groups completed the treatment for six months and contributed data to the analysis (RIC group, *n* = 18; control group, *n* = 19; Fig. 1).

The two treatment groups were similar in their baseline characteristics (Table 1), showing no significant differences in any of their characteristics before treatments started. The participants had high rates of hypertension (32/37, 86.5%) and diabetes mellitus (15/37, 40.5%). Most participants were concomitantly taking medications, with the most common being antihypertensive agents (29/37, 78.4%) and aspirin (21/37, 56.8%).

**Table 1:** Baseline Characteristics of Participants by Treatment Group

Characteristic	Treatment Group(*n* = 18)	Control Group(*n* = 19)	p
Age, mean (SD),y	67.6 (7.2)	70.6 (7.4)	0.216^a^
Female, n(%)	6 (33.3)	9 (47.4)	0.508^b^
Education, n(%),y			1.000^b^
≤5	1 (5.6)	2 (10.5)	
〉5	17 (94.4)	17 (89.5)	
Medical history, n(%)			
Hypertension	16 (88.9)	16 (84.2)	1.000^b^
Hyperlipidemia	5 (27.8)	5 (26.3)	1.000^b^
Diabetes mellitus	6 (33.3)	9 (47.4)	0.508^b^
Concomitant drugs, n(%)			
Antihypertensive agents	14 (77.8)	15 (78.9)	0.471^b^
Aspirin	11 (61.1)	10 (52.6)	0.527^b^
Lipid-reducing agents	5 (27.8)	5 (26.3)	0.651^b^
Hypoglycemic agents	6 (33.3)	8 (42.1)	0.621^b^
Psychometric scores, mean (SD)			
HAMD	5.06 ± 2.41	5.61 ± 3.76	0.913^c^
MMSE	23.44 ± 3.31	22.82 ± 3.99	0.883^c^
MOCA	18.28 ± 3.89	18.47 ± 4.78	0.807^c^
CDR	1.11 ± 0.58	1.27 ± 0.56	0.443^c^
HVLT1	12.56 ± 4.15	13.05 ± 5.33	0.869^c^
HVLT2	2.72 ± 2.61	2.53 ± 2.80	0.822^c^
HVLT3	6.22 ± 2.21	6.05 ± 2.44	0.845^c^
COWAT	5.11 ± 3.34	5.00 ± 3.32	0.852^c^
TMT-A	125.83 ± 69.54	147.47 ± 101.80	0.518^c^
TMT-B	262.83 ± 122.24	252.79 ± 118.28	0.916^c^
SDMT	19.67 ± 9.32	23.61 ± 15.34	0.542^c^
JLO	17.94 ± 7.60	17.50 ± 5.76	0.650^c^
ADL	25.33 ± 2.61	25.11 ± 2.18	0.799^c^
NPI	2.06 ± 1.11	1.74 ± 1.05	0.425^c^
Hs-CRP (mg/dL)	2.38 ± 2.87	1.63 ± 1.71	0.513 ^a^
IL-6 (pg/mL)	16.26 ± 7.05	16.25 ± 4.99	0.995^a^
TNF-α (pg/mL)	5.64 ± 1.12	5.54 ± 0.83	0.751^a^
Homocysteine	15.9 ± 6.34	15.6 ± 5.62	0.870 ^a^
Triglyceride (mmol/L)	1.89 ± 0.69	1.75 ± 0.44	0.487^a^
Cholesterol (mmol/L)	4.90 ± 1.12	4.52 ± 1.12	0.320^a^
LDL (mmol/L)	3.01 ± 0.87	2.63 ± 0.78	0.160^a^
HDL (mmol/L)	1.11 ± 0.29	1.29 ± 0.29	0.063^a^
WMLV (cm^3^)	84.00 ± 53.82	71.26 ± 28.03	0.663 ^a^
WBWM			
FA	0.30 ± 0.02	0.31 ± 0.02	0.28 ^a^
MD(×10^−4^)	9.5 ± 0.79	8.9 ± 0.33	0.22 ^a^
NAWM			
FA	0.30 ± 0.02	0.31 ± 0.17	0.315 ^a^
MD(×10^−4^)	8.9 ± 0.73	8.5 ± 0.37	0.389 ^a^

Abbreviations: *ADL* Activities of daily living, *CDR* Clinical Dementia Rating, *COWAT* Controlled Oral Word Association Test, the outcome was the sum of correct words in the both parts, *FA* fractional anisotropy, *HAMD* Hamilton Depression Scale, *hs-CRP* high-sensitive C-reactive protein, *HVLT1* immediate memory of Hopkins Verbal Learning Test-Revised (HVLT-R), *HVLT2* delayed memory of HVLT-R, HVLT3, recognition test of HVLT-R, *HDL* high density lipoprotein, *IL-6* interleukin 6, *JLO* Judgment of Line Orientation, *LDL* low density lipoprotein, *MD* mean diffusivity, *MMSE* Mini-Mental State Examination, *MOCA* Montreal Cognitive Assessment, *NAWM* normal-appearing white matter, *NPI* Neuropsychiatric Inventory, *SDMT* Symbol Digit Modalities Test, *TMT-A* Trail Making Test A, *TMT-B* Trail Making Test B, TNF-α, tumor necrosis factor-alpha, *WBWM* whole-brain white matter, *WMLV* white matter lesion volume

^a^Independent t-test

^b^Chi-square test or Fisher exact test

^c^ The Mann-Whitney U test

### Neuropsychological outcomes

Patients receiving repetitive RIC showed greater overall improvement in the battery of cognitive tests (HVLT-R, COWAT, TMT-A, TMT-B and JLO) after six months of treatment. By contrast, the control group showed less improvement, which was limited to HVLT-R and TMT-B (Table 2). The RIC group also showed greater improvement in the JLO at six months compared to the control group (RIC group 23.10 vs. control group 18.56; *P* = 0.013; Table 3).

**Table 2:** Efficacy Measures at 6 months

Efficacy Measure	RIC Group (*n* = 18)		p	Control Group (*n* = 19)		p
	Before	After		Before	After	
HVLT-R						
HVLT1	12.56 ± 4.15	16.89 ± 4.23	0.002	13.05 ± 5.33	16.05 ± 5.65	0.017
HVLT2	2.72 ± 2.61	4.72 ± 3.12	0.036	2.53 ± 2.80	4.53 ± 3.32	0.013
HVLT3	6.22 ± 2.21	8.83 ± 1.89	0.000	6.05 ± 2.44	7.42 ± 3.04	0.023
COWAT	5.11 ± 3.34	6.39 ± 3.33	0.030	5.00 ± 3.32	5.53 ± 3.89	0.455
TMT-A	125.83 ± 69.54	102.50 ± 57.33	0.007	131.88 ± 86.06	128.47 ± 112.45	0.848
TMT-B	262.83 ± 122.24	209.17 ± 108.98	0.015	244.61 ± 116.05	197.28 ± 133.79	0.007
SDMT	19.67 ± 9.32	21.17 ± 9.93	0.426	23.61 ± 15.34	21.72 ± 14.99	0.249
JLO	17.94 ± 7.60	23.22 ± 4.66	0.006	17.50 ± 5.76	18.44 ± 7.53	0.398
ADL	25.33 ± 2.61	24.72 ± 1.60	0.102	25.11 ± 2.18	24.84 ± 1.43	0.426
NPI	2.06 ± 1.11	1.72 ± 0.83	0.083	1.74 ± 1.05	1.53 ± 0.77	0.215
hs-CRP	2.38 ± 2.87	1.30 ± 1.35	0.221	1.63 ± 1.71	1.67 ± 1.80	0.951
IL-6	16.26 ± 7.05	15.85 ± 6.70	0.072	16.25 ± 4.99	15.81 ± 4.27	0.125
TNF-α	5.64 ± 1.12	5.57 ± 1.02	0.167	5.54 ± 0.83	5.46 ± 0.72	0.155
WMLV (cm^3^)	84.00 ± 53.82	69.87 ± 45.76	0.060	71.26 ± 28.03	68.35 ± 23.20	0.569
WBWM						
FA	0.30 ± 0.02	0.29 ± 0.02	0.348	0.31 ± 0.02	0.30 ± 0.04	0.446
MD(×10^−4^)	9.5 ± 0.79	9.6 ± 0.84	0.128	8.9 ± 0.33	9.1 ± 0.62	0.288
NAWM						
FA	0.30 ± 0.02	0.30 ± 0.02	0.293	0.31 ± 0.02	0.30 ± 0.04	0.427
MD(×10^−4^)	9.0 ± 0.73	9.4 ± 0.60	0.136	8.6 ± 0.37	8.9 ± 0.60	0.097

Abbreviations: *ADL* Activities of daily living, *COWAT* Controlled Oral Word Association Test, the outcome was the sum of correct words in the both parts, *FA* fractional anisotropy, *hs-CRP* high-sensitive C-reactive protein, *HVLT1* immediate memory of Hopkins Verbal Learning Test-Revised (HVLT-R), *HVLT2* delayed memory of HVLT-R, *HVLT3* recognition test of HVLT-R, *IL-6* interleukin 6, *JLO* Judgment of Line Orientation, *MD* mean diffusivity, *NAWM* normal-appearing white matter, *NPI* Neuropsychiatric Inventory, *SDMT* Symbol Digit Modalities Test, *TMT-A* Trail Making Test A, *TMT-B* Trail Making Test B, *TNF-α* tumor necrosis factor-alpha, *WBWM* whole-brain white matter, *WMLV* white matter lesion volume

Notes: Paired t-test was used to evaluate the effect of the treatments in the group

**Table 3:** Covariance analysis of efficacy between the Two Groups

Psychometric scores	RIC Group(*n* = 18)	Control Group(*n* = 19)	p
HVLT-R			
HVLT1	17.02 ± 1.05	15.93 ± 1.02	0.464
HVLT2	4.68 ± 0.73	4.56 ± 0.71	0.908
HVLT3	8.78 ± 0.50	7.47 ± 0.48	0.069
COWAT	6.35 ± 0.62	5.57 ± 0.60	0.372
TMT-A	105.12 ± 13.09	125.70 ± 13.47	0.282
TMT-B	201.64 ± 17.29	204.80 ± 17.29	0.898
SDMT	22.82 ± 1.68	20.07 ± 1.68	0.259
JLO	23.10 ± 1.23	18.56 ± 1.23	0.013
Hs-CRP	2.63 ± 0.74	1.19 ± 0.83	0.220
IL-6	15.85 ± 6.70	15.81 ± 4.27	0.790
TNF-α	5.57 ± 1.02	5.46 ± 0.72	0.679
WMLV (cm^3^)	74.21 ± 4.24	65.18 ± 3.79	0.166
WBWM			
FA	0.291 ± 0.009	0.306 ± 0.009	0.336
MD(×10^−4^)	9.5 ± 0.32	9.0 ± 0.71	0.658
NAWM			
FA	0.292 ± 0.01	0.307 ± 0.01	0.358
MD(×10^−4^)	9.3 ± 0.46	8.9 ± 0.62	0.485

Abbreviations: *COWAT* Controlled Oral Word Association Test, the outcome was the sum of correct words in the both parts, *FA* fractional anisotropy, *hs-CRP* high-sensitive C-reactive protein, *HVLT1* immediate memory of Hopkins Verbal Learning Test-Revised (HVLT-R), *HVLT2* delayed memory of HVLT-R, *HVLT3* recognition test of HVLT-R, *IL-6* interleukin 6, *JLO* Judgment of Line Orientation, *MD* mean diffusivity, *NAWM* normal-appearing white matter, *SDMT* Symbol Digit Modalities Test, *TMT-A* Trail Making Test A, *TMT-B* Trail Making Test B, *TNF-α* tumor necrosis factor-alpha, *WBWM* whole-brain white matter, *WMLV* white matter lesion volume

### Inflammatory factors

The level of plasma hs-CRP, IL-6 and TNF-α did not differ significantly in the two groups before and after treatment, although the RIC group exhibited a non-significant downward trend after treatment (Table 2).

### MRI

Although the RIC group showed a tendency for improvement in WMLV after treatment (RIC group, pre-treatment 84.00 vs. post-treatment 69.87, *P* = 0.060; control group, 71.26 vs. 68.35, *P* = 0.569) (Table 2), the WMLV showed no significant differences between the two groups at the six month follow up (*P* = 0.166) (Table 3).

When comparing the parameters before and after treatment within the group, DTI results revealed that the FA and MD parameters of the WBWM/NAWM did not change significantly(*P〉0.05)* (Table 2). The comparison of the FA and MD parameters before and after treatment between those two groups also showed no significant differences. (Table 3).

### Safety

All patients completed the treatment without incident. There were no obvious adverse effects, such as venous congestion, bleeding, etc., Only one patient in the RIC group complained of mild skin reactions, which disappeared after putting a towel between the cuff and his skin.

## Discussion

SIVD is the most common type of vascular dementia, but unfortunately there is no effective treatment to date. Hence, other therapeutic approaches are needed for these patients, which might bring them a convenient intervention for relief or slow down their cognitive exacerbation. Researches showed that RIC could reduce the recurrence of large atherosclerotic cerebral infarction by stabilizing plaque, increasing cerebral blood flow and reducing the release of pro-inflammatory factors, etc. [6, 19]. Multiple studies showed reduced cerebral perfusion in patients with small vessel disease [20, 21]. Cerebral small vessel disease is associated with cognitive impairment, due to the decreased microvascular perfusion and microstructural integrity [8, 22, 23]. SIVD is characterized by cerebral small vessel incomplete occlusion or lacunar infarction, and accompanied by destruction of blood-brain barrier and invasion of inflammation [2]. Therefore, we further explore the therapeutic effect of RIC on patients with SIVD,

The previous study showed that RIC may be effective in patients with cerebral small-vessel disease-related mild cognitive impairment [24]. As evidenced by the improvement in JLO performance, our study indicates that RIC may also improve visuospatial perception and spatial orientation ability in patients with SIVD. No significant group difference in treatment efficacy was observed in other cognitive domains. However, the improvement of cognitive performance in the RIC group was more comprehensive than that in the control group. Participants in the RIC group performed significantly better on 4 of the 5 cognitive tests six months after treatment, whereas participants in the control group performed significantly better on 2 of the 5. These results suggest that RIC is an effective treatment for staving off, or modifying the cognitive decline associated with SIVD.

Several pathogenic mechanisms, including molecular inflammation, hypoperfusion, and structural anatomical network disruption may converge to cause SIVD [25]. Research shows that vascular dementia risk increases with increasing hs-CRP and the size of WMLV is associated with cognitive decline [26, 27]. RIC has the effect of anti-inflammation and increasing cerebral blood flow [6, 28]. Although no significant difference was observed in hs-CRP/IL-6/TNF-α plasma levels and WMLV before and after treatment, absolute hs-CRP/IL-6/TNF-α levels and WMLV showed a more pronounced tendency to decrease in the RIC group.

DTI has been used to detect possible disconnection or anatomical disruption of brain circuits and shows stronger correlations with cognition than with WMLV [29]. Diseases that disrupt the directionality of fiber tracts and white matter integrity show reduced FA and increased MD [30]. Loss of microstructural integrity of white matter is related to cognitive disturbances, which mainly localizes to NAWM [31]. The present study of the MRI results failed to show any change in white matter brain circuits after RIC. It might be attributed to the limitation of the observation period, which may not be long enough for detection of the radiological changes in the brain structure. The biological markers of CRP and WMLV involved in this study didn’t show significant differences after the RIC treatment, however, the trend of the changes encouraged us to further investigate more biological markers like Arterial Spin Labeling for prediction and evaluation of the therapeutic effect in SIVD. This study gives us clue for further clinical trials with increased sample size regarding potential biological markers related to the outcome of RIC in SIVD.

A consistent theme throughout our study was that participants receiving RIC tended to perform better on the cognitive tests than participants receiving sham RIC (control group). Surprisingly, cognitive function in the control group did not decline much over the six-month assessment period. In some cases, they even appeared to improve in some cognitive domains (HVLT-R and TMT-B). The absence of much cognitive decline in the control group may be related to the following factors: (1) the combined treatment of medicine reduced the occurrence of ischemic events and delayed the progression of cognitive impairment; (2) SIVD was at a slowly progressing stage, and a longer time period may be necessary to detect significant cognitive decline; (3) we did not strictly limit patients using citicoline or *Ginkgo biloba* leaves extract tablets, which could have exerted nootropic effects in a small number of patients; (4) The sham treatment with inflation to 60 mmHg maybe also have effect of mild ischemic conditioning; (5) the presence of a placebo effect may elevate performance to some degree.

Although our study suggests that RIC is a feasible positive treatment strategy for SIVD, several limitations of the study must be considered. First, the number of the patients was small, which reduced our statistical power. Although the patients with other causes of dementia were excluded, the participants with mixed dementia could not be excluded completely. Second, the observational period was short and lasted only 6 months, which might not be long enough to observe the effect of RIC. Finally, although our study found improvement in clinical outcomes related to cognition, changes in biomarker levels and imaging markers were not significant. Thus, we cannot confidently suggest any possible mechanisms that RIC may have on the pathogenesis of SIVD. In addition to the MRI parameters, studies reported the significant treatment effects in cerebral blood flow velocities and pulsatile index, indicating better cerebral hemodynamic status after RIC [32]. In further studies, we will use cerebral blood flow such as Transcranial Doppler (TCD), Arterial Spin Labeling (ASL) to evaluate the efficacy.

## Conclusions

This study suggests that repetitive RIC treatment for six months is a feasible and potentially effective way to improve cognition in patients with SIVD. Further trials using a longer duration of RIC treatment and larger sample sizes are warranted.

## Data Availability

The datasets used or analysed during the current study are available from the corresponding author on reasonable request.

## Abbreviations

ADL: Activities of daily living
CDR: Clinical Dementia Rating
COWAT: Controlled Oral Word Association Test, the outcome was the sum of correct words in the both parts
DTI: diffusion tension imaging
FA: fractional anisotropy
HAMD: Hamilton Depression Scale
HDL: high density lipoprotein
hs-CRP: high-sensitive C-reactive protein
HVLT1: immediate memory of Hopkins Verbal Learning Test-Revised (HVLT-R)
HVLT2: delayed memory of HVLT-R
HVLT3: recognition test of HVLT-R
IL-6: interleukin 6
JLO: Judgment of Line Orientation
LDL: low density lipoprotein
MD: mean diffusivity
MMSE: Mini-Mental State Examination
MOCA: Montreal Cognitive Assessment
MRI: MR imaging
NAWM: normal-appearing white matter
NPI: Neuropsychiatric Inventory
RIC: remote ischemic conditioning
SDMT: Symbol Digit Modalities Test
SIVD: Subcortical ischemic vascular dementia
TMT-A: Trail Making Test A
TMT-B: Trail Making Test B
TNF-α: tumor necrosis factor-alpha
WBWM: whole-brain white matter
WML: white matter lesion
WMLV: white matter lesion volume
